# The formation of diploid and triploid hybrids of female grass carp × male blunt snout bream and their 5S rDNA analysis

**DOI:** 10.1186/1471-2156-14-110

**Published:** 2013-11-23

**Authors:** Weiguo He, Lihua Xie, Tangluo Li, Shaojun Liu, Jun Xiao, Jie Hu, Jing Wang, Qinbo Qin, Yun Liu

**Affiliations:** 1 Key Laboratory of Protein Chemistry and Fish Developmental Biology of Education Ministry of China, College of Life Sciences, Hunan Normal University, Changsha 410081, P R. China

**Keywords:** Hybridization, Ployploidization, Fish genetic breeding, 5S rDNA

## Abstract

**Background:**

Hybridization is a useful strategy to alter the genotypes and phenotypes of the offspring. It could transfer the genome of one species to another through combing the different genome of parents in the hybrid offspring. And the offspring may exhibit advantages in growth rate, disease resistance, survival rate and appearance, which resulting from the combination of the beneficial traits from both parents.

**Results:**

Diploid and triploid hybrids of female grass carp (*Ctenopharyngodon idellus*, GC, Cyprininae, 2n = 48) × male blunt snout bream (*Megalobrama amblycephala*, BSB, Cultrinae, 2n = 48) were successfully obtained by distant hybridization. Diploid hybrids had 48 chromosomes, with one set from GC and one set from BSB. Triploid hybrids possessed 72 chromosomes, with two sets from GC and one set from BSB.

The morphological traits, growth rates, and feeding ecology of the parents and hybrid offspring were compared and analyzed. The two kinds of hybrid offspring exhibited significantly phenotypic divergence from GC and BSB. 2nGB hybrids showed similar growth rate compared to that of GC, and 3nGB hybrids significantly higher results. Furthermore, the feeding ecology of hybrid progeny was omnivorous.

The 5S rDNA of GC, BSB and their hybrid offspring were also cloned and sequenced. There was only one type of 5S rDNA (designated type I: 180 bp) in GC and one type of 5S rDNA (designated type II: 188 bp) in BSB. However, in the hybrid progeny, diploid and triploid hybrids both inherited type I and type II from their parents, respectively. In addition, a chimera of type I and type II was observed in the genome of diploid and triploid hybrids, excepting a 10 bp of polyA insertion in type II sequence of the chimera of the diploid hybrids.

**Conclusions:**

This is the first report of diploid and triploid hybrids being produced by crossing GC and BSB, which have the same chromosome number. The obtainment of two new hybrid offspring has significance in fish genetic breeding. The results illustrate the effect of hybridization and polyploidization on the organization and variation of 5S rDNA in hybrid offspring.

## Background

The term hybrid can be defined as organisms formed by cross-fertilization between individuals of different species, or it can be applied more broadly to the offspring between individuals from populations which are distinguishable on the basis of one or more heritable characters [1]. Hybridization can influence evolution in a variety of ways, and the hybrid offspring may have heterosis to their parents [2]. It is generally thought that hybridization plays an important role in triggering polyploidization [3-6]. Distant hybridization is a useful strategy to generate polyploids in fish, and the approach results in transfer of the genome of one species to another. This leads to alteration of the genotypes and phenotypes of the offspring. A combination of the beneficial traits from both parents often results in the hybrids possessing the advantages of improved appearance, faster growth rate, higher survival and disease resistance [7]. In addition, many artificially induced polyploid fish have been used in aquaculture to produce sterility and to improve production [8-10]. Furthermore, teleost polyploids are a useful model system to verify the theories about the origin and consequences of polyploidization [11].

Grass carp have been introduced throughout the world because of their value as a food fish and their ability to control aquatic vegetation. In addition, grass carp grow rapidly compared to other common fish [12,13]. By using GC (*Ctenopharyngodon idellus*) and BSB (*Megalobrama amblycephala*), diploid and triploid hybrid fish were successfully produced. It is the first time to obtain the diploid and triploid hybrids by crossing GC and BSB which possess the same chromosome number in vertebrates.

Accumulation of information about the genomic organization of fish is related to the repetitive portion of their genome, of which considerable attention has focused on rDNA. In eukaryotes, tandem arrays of rDNA are organized into two distinct gene families, including a major family that encodes for 28S, 5.8S and 18S rDNA and a minor family that encodes for 5S rDNA. The 5S rDNA multigene family is composed of a highly conserved 5S rRNA sequence of 120 bp and a variable nontranscribed spacer (NTS) which form arrays of hundreds to thousands of tandem repeats [14-16]. The 5S rRNA sequences are highly conserved in length and nucleotide sequence, even among non-related taxa, and there is a high rate of variation in the NTS sequences, which are species-specific [17,18]. Although a number of studies have reported the structural and functional organization of the 5S rDNA arrays in teleosts [16,19-25], relatively few have done so for hybrids [18,26,27].

To improve our understanding of the dynamics of 5S rDNA tandem repeats in hybrid fish and document the impact of hybridization and polyploidzation on 5S rDNA organization, we conducted an analysis of nucleotide sequences and molecular organization of 5S rDNA in grass carp (GC), blunt snout bream (BSB) and their hybrid offspring.

## Results

### Formation of diploid and triploid hybrids

The crossing of GC♀ × BSB♂ resulted in a high fertilization rate (95%) and hatching rate (80%) but low adulthood rate (1%). Approximately 200 2nGB and 800 3nGB hybrids were obtained each year.

### Chromosome number and karyotypes

**Figure 1:**
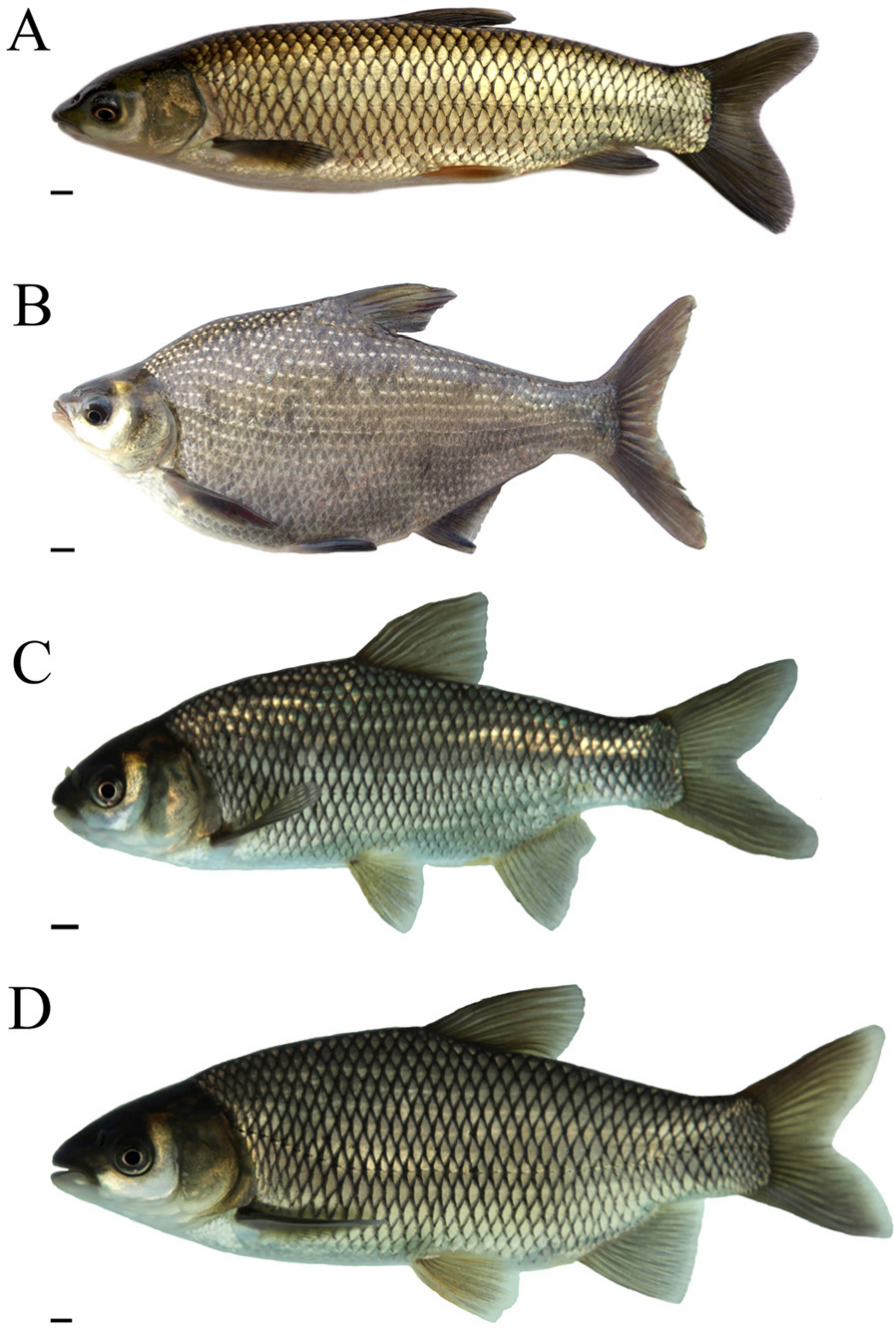
**Appearance of GC**, **BSB and their hybrid offspring. (A)** GC. **(B)** BSB. **(C)** The 2nGB hybrids of GC × BSB. **(D)** The 3nGB hybrids of GC × BSB. Bar in **A-D**, 1 cm.

**Figure 2:**
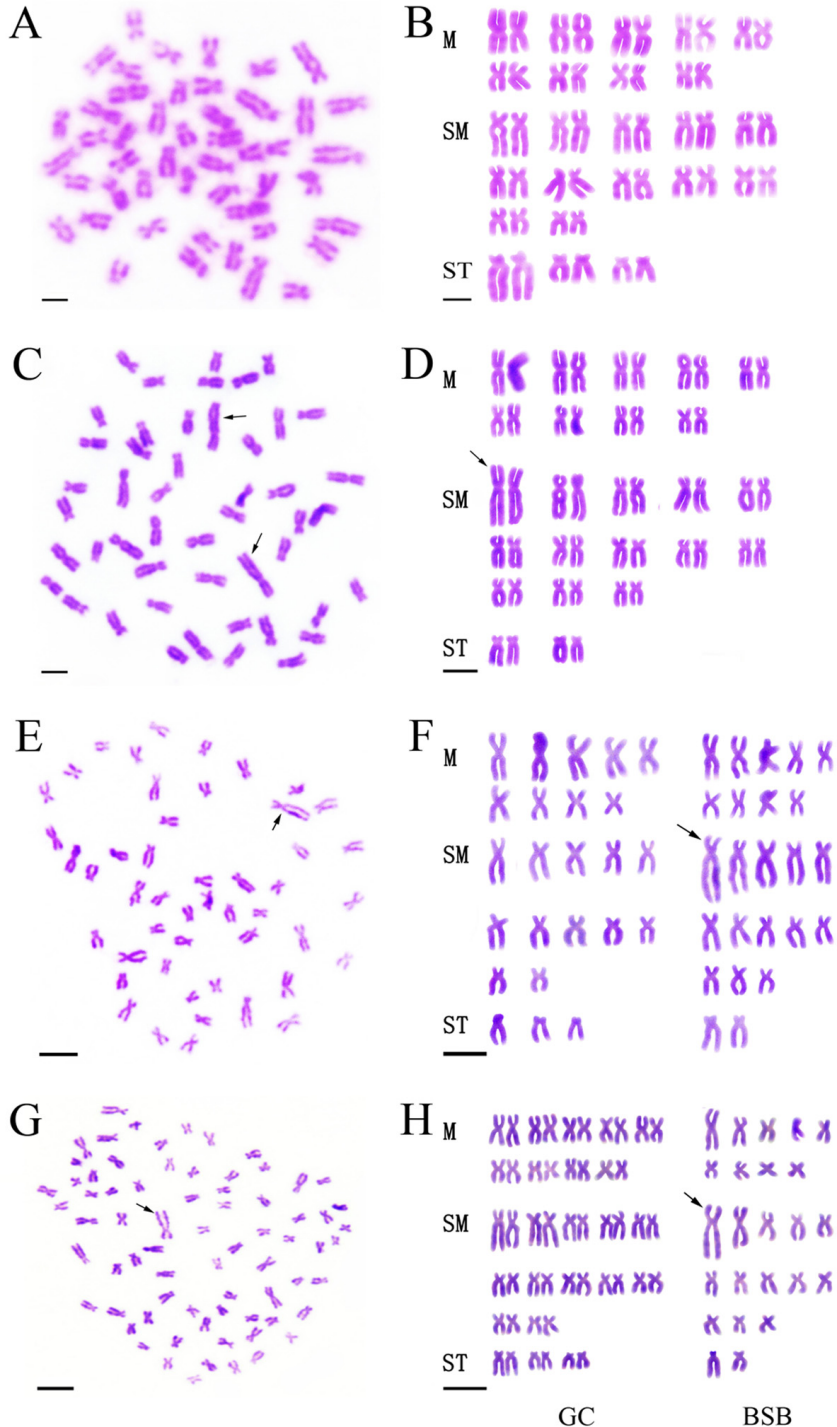
**Chromosome spreads at metaphase and corresponding karyotypes of GC**, **BSB and their hybrid offspring. (A)** The 48 chromosomes of GC, with no large submetacentric chromosome. **(B)** The karyotype of GC, in which no large submetacentric chromosome is detected. **(C)** The 48 chromosomes of BSB, with a pair of the largest submetacentric chromosomes indicated (solid arrows). **(D)** The karyotype of BSB, which includes a pair of the largest submetacentric chromosomes (solid arrow). **(E)** The 48 chromosomes of 2nGB hybrids, with a piece of the largest submetacentric chromosome indicated (solid arrow). **(F)** The karyotype of 2nGB hybrids, comprising one set of chromosomes from GC and one set from BSB. The solid arrow indicates a piece of the largest submetacentric chromosome, which is similar to that of BSB. **(G)** The 72 chromosomes of 3nGB hybrids, with a piece of the largest submetacentric chromosome indicated (solid arrow). **(H)** The karyotype of 3nGB hybrids, consisting of two sets of chromosomes from GC and one set from BSB. The solid arrow indicates a piece of the largest submetacentric chromosome, which is similar to that of BSB. Scale bar in **A–H**, 3 μm.

The distribution of chromosome numbers in GC, BSB, and the 2nGB and 3nGB hybrids is given in Table 1. For diploid GC (Figure 1A), 94.5% of chromosomal metaphases had 48 chromosomes with the karyotype formula of 18m + 24sm + 6st (Figure 2A, B). For diploid BSB (Figure 1B), 95.5% of chromosomal metaphases possessed 48 chromosomes with the karyotype formula of 18m + 26sm + 4st (Figure 2C, D). In the hybrid offspring of GC♀ × BSB♂ with low body height (Figure 1C), 92.5% of chromosomal metaphases had 48 chromosomes with the karyotype formula of 18m + 25sm + 5st (Figure 2E, F). In the hybrid offspring of GC♀ × BSB♂ having high body height (Figure 1D), 91.5% of chromosomal metaphases had 72 chromosomes with the karyotype formula of 27m + 37sm + 8st (Figure 2G, H).

**Table 1:** **Chromosome number in GC**, **BSB**, **and 2nGB and 3nGB hybrids**

**Fish type**	**No. in metaphase**	**Distribution of chromosome number**			
		<**48**^**a**^	**48**	<**72**^**a**^	**72**
GC	200	11	189		
BSB	200	9	191		
2nGB	200	15	185		
3nGB	200			17	183

^a^ The chromosome number is less than what they should be, owning to the loss of chromosomes in the procedure of chromosome preparation.

### DNA content

**Figure 3:**
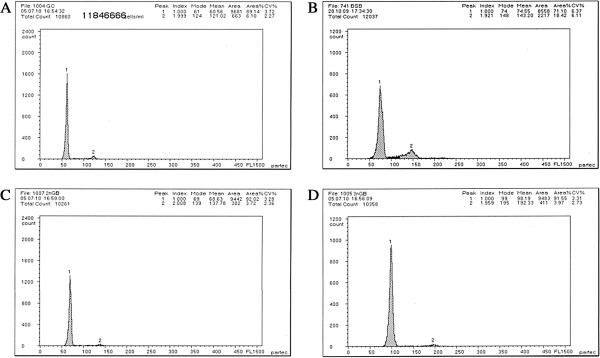
**Cytometric histograms of GC**, **BSB and their hybrid offspring. (A)** The mean DNA content of GC (peak 1: 60.56). **(B)** The mean DNA content of BSB (peak 1: 74.55). **(C)** The mean DNA content of 2nGB (peak 1: 68.63). **(D)** The mean DNA content of 3nGB (peak 1: 98.19).

The distribution of DNA content of all samples is given in Table 2 and Figures 3A–D. The mean DNA content of 2nGB hybrids was equal (*P* > 0.05) to the sum of half of GC and BSB, suggesting that 2nGB hybrids had one set of chromosomes from GC and BSB, respectively. The mean DNA content of 3nGB hybrids was equal (*P* > 0.05) to the sum of GC and half of BSB, suggesting that 3nGB hybrids had two sets of chromosomes from GC and one set of chromosomes from BSB.

**Table 2:** **Mean DNA content in GC**, **BSB**, **and 2nGB and 3nGB hybrids**

**Fish type**	**Mean **^**a **^**DNA content**	**Ratio**	
		**Observed**	**Expected**
GC	60.56		
BSB	74.55		
2nGB	68.63	2nGB/(0.5 GC + 0.5 BSB) = 1.02^b^	1
3nGB	98.19	3nGB/(GC + 0.5 BSB) = 1.00^b^	1

^a^The intensity of fluorescence (unit, channel).

^b^The observed ratio was not significantly different (*P* > 0.05) from the expected ratio.

### Morphological traits

The countable and measurable trait values for GC, BSB, and their hybrid offspring are given in Tables 3 and 4. There was a significant difference in the countable traits between 2nGB hybrids and GC, except for the number of abdominal fins (*P* > 0.01). Similarly, there was a significant difference in all countable traits between 2nGB hybrids and BSB, between 3nGB hybrids and GC, and between 3nGB hybrids and BSB. The countable traits, except for the number of upper lateral scales, differed significantly between 2nGB and 3nGB hybrids.

**Table 3:** **Comparison of the countable traits between the different ploidy**-**level hybrids and their parents**

**Fish type**	**No. of lateral scales**	**No. of upper lateral scales**	**No. of lower lateral scales**	**No. of dorsal fins**	**No. of abdominal fins**	**No. of anal fins**
GC	41.10 ± 1.20(39 ~ 42)	6.40 ± 0.52(6 ~ 7)	4.60 ± 0.52(4 ~ 5)	III + 7.00 ± 0.00(III + 7)	8.00 ± 0.00(8)	III + 7.00 ± 0.00(III + 7)
BSB	50.90 ± 1.20(49 ~ 52)	9.50 ± 0.53(9 ~ 10)	10.10 ± 0.99(9 ~ 11)	III + 8.50 ± 0.53(III + 8 ~ 9)	9.20 ± 0.63(8 ~ 10)	III + 25.90 ± 0.88(III + 25 ~ 27)
2nGB	46.50 ± 0.53(46 ~ 47)	8.00 ± 0.00(8)	6.00 ± 0.00(6)	III + 8.00 ± 0.00(III + 8)	8.00 ± 0.00(8)	III + 15.70 ± 0.48(III + 15 ~ 16)
3nGB	45.00 ± 1.05(43 ~ 46)	8.00 ± 0.00(8)	5.60 ± 0.52(5 ~ 6)	III + 9.00 ± 0.00(III + 9)	8.60 ± 0.52(8 ~ 9)	III + 13.40 ± 0.84(III + 12 ~ 14)

Uppercase Roman numerals indicate the number of the spines, and Arabic numerals reflect the number of soft fins. Numbers before and after the symbol “±” present the mean value and standard deviation of numbers of the fins respectively. “~” implies the measured number range of the fins.

**Table 4:** Comparison of the measurable traits between the hybrid offspring and their parents

**Fish type**	**BL/WL**	**BW/BL**	**HL/BL**	**HW/HL**	**TW/TL**	**HW/BW**
GC	0.84 ± 0.04	0.26 ± 0.04	0.25 ± 0.04	0.77 ± 0.03	0.86 ± 0.03	0.73 ± 0.03
BSB	0.84 ± 0.03	0.42 ± 0.03	0.21 ± 0.04	0.88 ± 0.03	0.93 ± 0.04	0.48 ± 0.04
2nGB	0.83 ± 0.01	0.30 ± 0.05	0.23 ± 0.01	0.74 ± 0.06	0.78 ± 0.07	0.58 ± 0.04
3nGB	0.83 ± 0.02	0.27 ± 0.01	0.22 ± 0.02	0.81 ± 0.08	0.93 ± 0.07	0.67 ± 0.06

There was a significant difference in the measureable traits between 2nGB hybrids and GC, except for the ratio of BL/WL which was not significantly different (*P* > 0.01). Except for the ratio of BL/WL and BW/BL there was a significant difference in the remaining measurable traits between 3nGB hybrids and GC. There was a significant difference in all measurable traits between 2nGB hybrids and BSB. Last, except for the ratios of BL/WL, HL/BL, and TW/TL, all other ratios differed significantly between 3nGB hybrids and BSB.

### Growth rate and feeding ecology

When cultured under the same conditions for one year, GC and 2nGB hybrids grew at a similar rate, and 3nGB hybrids presented the fastest rate. The mean weight for GC, 2nGB and 3nGB hybrids was 1.56 kg, 1.58 kg and 4.22 kg, respectively, and the maximum was 1.67 kg, 1.65 kg and 4.75 kg, respectively. The growth rate of 2nGB hybrids was 1.01 times faster than that of GC, and the growth rate of 3nGB hybrids was 2.71 times faster than that of GC and 2.67 times faster than that of 2nGB hybrids (Table 5). Similar to BSB, GC was herbivorous. However, 2nGB and 3nGB hybrids were omnivorous.

**Table 5:** **Comparisons of growth rates of GC**, **and 2nGB hybrids and 3nGB hybrids** (**unit**, **kg**)

	**January**	**February**	**March**	**April**	**May**	**June**	**July**	**August**	**September**	**October**	**November**	**December**
GC	0.45	0.49	0.54	0.58	0.66	0.75	0.9	1.04	1.21	1.35	1.48	1.56
2nGB	0.47	0.50	0.53	0.58	0.68	0.80	0.94	1.09	1.24	1.37	1.49	1.58
3nGB	1.28	1.34	1.46	1.65	1.89	2.18	2.53	2.95	3.31	3.66	3.97	4.22

### Molecular organization of 5S rDNA

**Figure 4:**
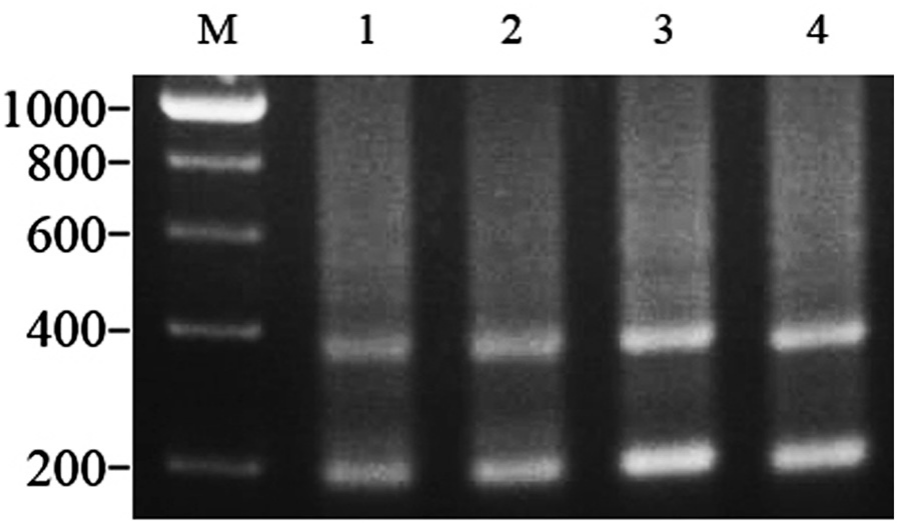
**DNA bands amplified from GC**, **BSB**, **and 2nGB and 3nGB hybrids.** M, DNA ladder markers (200 bp increments); lane 1, two DNA bands (~200 and 400 bp respectively) from GC; lane 2, two DNA bands (~200 and 400 bp respectively) from BSB; lane 3, two DNA bands (~200 and 400 bp respectively) from 2nGB hybrids; lane 4, two DNA bands (~200 and 400 bp respectively) from 3nGB hybrids.

Using the 5S primer pair, the agarose gel electrophoresis band patterns (approximately 200 and 400 bp, respectively) amplified from GC, BSB, 2nGB and 3nGB hybrids were similar (Figure 4). To further evaluate differences of 5S rDNA patterns, a total of 160 clones were cloned, including 40 clones from GC, 40 clones from BSB, 40 clones from 2nGB hybrids and 40 clones from 3nGB hybrids, (see details in Table 6). There were two different sizes (180 and 360 bp) in GC, two in BSB (188 and 376 bp), four in 2nGB hybrids (180, 188, 360 and 378 bp), and four in 3nGB hybrids (180, 188, 360 and 368 bp) (Table 6). Sequencing analysis indicated that 2nGB hybrids had two similarly sized 5S rDNA fragments (180 and 188 bp, 360 and 378 bp) that were not distinguishable on the agarose gel, where they were seen as single bands of about 200 and 400 bp, respectively. The results were similar for 3nGB hybrids.

**Table 6:** The results of 5S sequencing

**Samples**	**Number of sequenced clones**	**PCR bands**	
		**~200 bp**^**a**^	**~400 bp**^**a**^
GC	40	20 clones of 180 bp	20 clones of 360 bp
BSB	40	20 clones of 188 bp	20 clones of 376 bp
2nGB	40	12 clones of 180 bp; 8 clones of 188 bp	6 clones of 360 bp; 14 clones of 378 bp
3nGB	40	9 clones of 180 bp; 11clones of 188 bp	8 clones of 360 bp; 12 clones of 368 bp

^**a**^The approximate size of PCR bands on the agarose gel.

**Figure 5:**
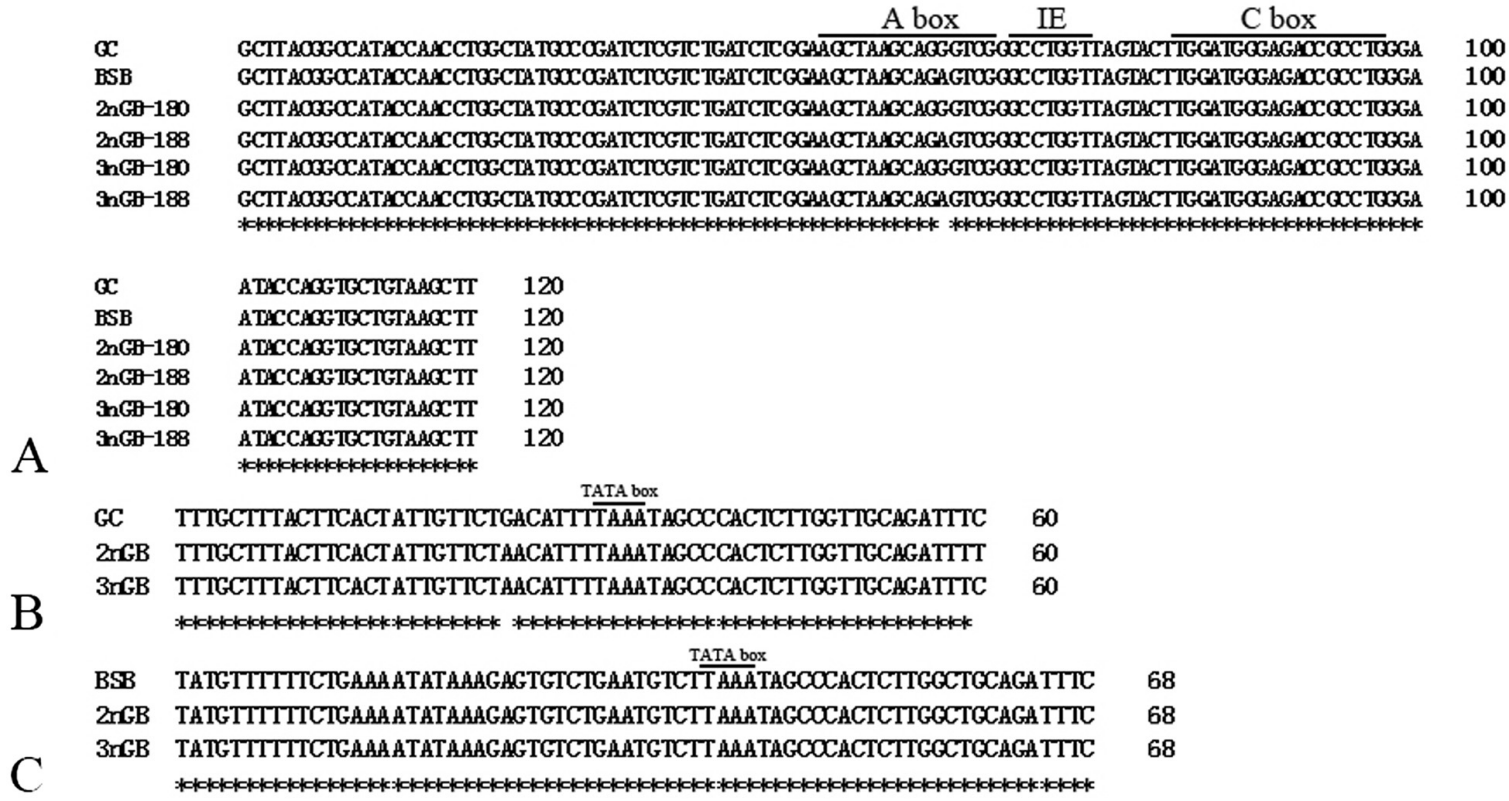
**Comparison of the 5S coding regions and the comparison of NTS sequences from GC**, **BSB and their hybrid offspring. (A)** Complete 5S coding regions from GC, BSB and their hybrid offspring; **(B)** The 60 bp NTS sequences from GC, and 2nGB and 3nGB hybrids; **(C)** The 68 bp NTS sequences from BSB, and 2nGB and 3nGB hybrids. Asterisks indicate the consensus nucleotides.

**Figure 6:**
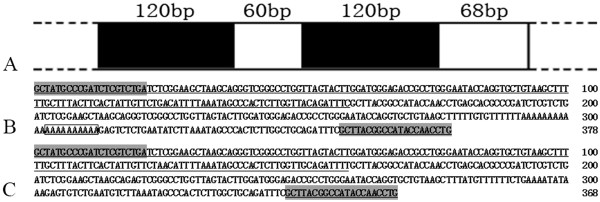
**The mosaic organization of 5S rDNA multigene families in 2nGB and 3nGB hybrids. A**, The illustrative diagram of the mosaic organization of 5S rDNA; **B**, Mosaic 5S rDNA tandem arrays from 2nGB hybrids; **C**, Mosaic 5S rDNA tandem arrays from 3nGB hybrids. The monomeric 5S rDNA from GC is underlined and the primer-combined regions are shaded. The insertion of poly A was included in the box.

**Figure 7:**
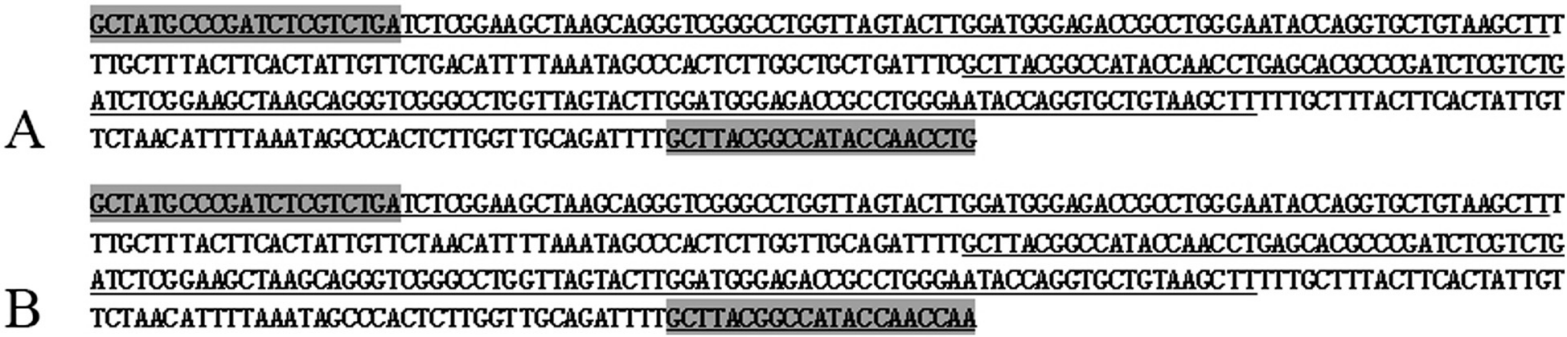
**Dimeric 5S rDNA tandem arrays of hybrid offspring. (A)** 2nGB hybrids; **(B)** 3nGB hybrids. The 5S coding sequence is underlined and the primer-combined regions are shaded.

Based on the BLASTn analysis, all fragments from GC, BSB, 2nGB and 3nGB hybrids were confirmed as 5S rDNA repeat units, consisting of a complete 5S rRNA region and a whole NTS region (Figure 5). In GC, only one fragment of 5S rDNA (designated type I: 180 bp) was characterized by the NTS sequence (designated NTS-I for the 60 bp monomers). In BSB, only one fragment of 5S rDNA (designated type II: 188 bp) was characterized by the NTS sequence (designated NTS-II for the 68 bp monomers). The 360 bp DNA fragments of GC and 376 bp DNA fragments of BSB were dimeric 5S rDNA tandem arrays consisting of two type I sequences and two type II sequences, respectively (data not show). These sequencing results suggest that GC and BSB are highly conserved in the 5S RNA regions but exhibit large variation in the NTS regions. A comparative analysis of homology of the 5S rDNA fragments between hybrid offspring and their parents indicated that 2nGB hybrids not only inherited type I and type II from their parents (GC and BSB respectively), but also generated a chimera of type I and type II, in which a 10 bp poly A was inserted in the type II sequence (Figure 6A, B). 3nGB hybrids harbored the similar situation to that of 2nGB hybrids (Figure 6A, C), but no insertion of poly A. In addition, the 360 bp DNA fragments from 2nGB hybrids and 3nGB hybrids were dimeric 5S rDNA tandem arrays consisting of two type I sequences (Figure 7).

A box, internal element (IE) and C box, constituting the internal control region (ICRs), were all identified in the 5S rRNA regions of GC, BSB and their hybrid offspring. Furthermore, all the ICRs were highly conserved. Nonetheless, a species-specific nucleotide site within A box (positioned 60) was yet observed between GC and BSB (Figure 5A).

There was only one type of NTS sequence in both GC and BSB, designated as NTS-I (60 bp) and NTS-II (68 bp), respectively. In NTS-I, GC, 2nGB and 3nGB hybrids showed a high homology among them, excepting a transition at position −1 (C → T) in 2nGB hybrids and a transition at position −36 (G → A) in 2nGB and 3nGB hybrids. The NTS-II sequence was the same between BSB and 2nGB and 3nGB hybrids. The TATA box control element, located at position −26 to −29 where it has been modified to TAAA, was observed within all of the NTS sequences of GC, BSB and their hybrid offspring. In addition, the thymidine residues, which are required for transcription termination, were also detected (Figure 5B, C).

## Discussion

Both the chromosome number and karyotype formula of BSB were consistent with previous reports [10]. In addition, a pair of the largest submetacentric chromosomes can be used as marker chromosomes for identifying BSB from GC. With a submetacentric largest chromosome, we confirmed that 2nGB hybrids contain one set of chromosomes from GC and one set of chromosomes from BSB (Figure 2E, F). Possessing a submetacentric largest chromosome, 3nGB hybrids apparently obtained two sets of chromosomes from GC and one set of chromosomes from BSB (Figure 2G, H). In addition, we detected a significant relationship between the sum of the mean DNA content of the hybrid offspring and their parents (Table 2, Figure 3). Furthermore, the morphological traits (including measurable and countable traits) of 2nGB and 3nGB hybrids were intermediate between GC and BSB. Our results provide support for the hybridization origin of the offspring of GC♀ × BSB♂, rather than the outcome of gynogenesis or androgenesis.

Prior studies have concluded that diploid or triploid hybrids are more likely to form than tetraploid hybrids when the parents have the same chromosome number in the first generation of the distant hybridization [7]. In this study, for the first time, by crossing different parents (GC and BSB), which belong to distinct subfamilies with the same chromosome numbers, diploid and triploid hybrids (2nGB and 3nGB) were successfully obtained. In our previous research, only diploid hybrids (2n = 100) were produced in the F_1_-F_2_ generation by crossing female red crucian carp (*Carassius auratus* red var., RCC) and male common carp (*Cyprinus carpio*, CC) which both possess 100 chromosomes, and the tetraploid hybrids (4nRT) were not observed until F_3_ and later generations [9]. Bisexual and fertile 2nGB hybrids, following generations of genetic breeding, were expected to be obtained in further studies.

Distant hybrid not only results in different ploidy levels of hybrid offspring, but also leads to phenotypic changes of the hybrids. The number of countable traits in 2nGB and 3nGB hybrids presented the intermediate range compared to that of GC and BSB (Table 3). The number of measurable traits in 2nGB hybrids was beyond the range in HW/HL and TW/TL compared to that of their parents, and 3nGB hybrids were closer in TW/TL to that of BSB (Table 4). The average growth rate of 2nGB hybrids was similar to that of GC, while 3nGB hybrids greater (Table 5). Moreover, 2nGB and 3nGB hybrids were omnivorous, suggesting that the hybrid offspring would be more adaptable to a range of diets during culture. In a word, theses two hybrid offspring present phenotypic divergence and hybrid heterosis from GC and BSB.

The structural and functional organization of the 5S rRNA genes have been described for fungi [28-31], plants [32,33] and animals [15,19-25,34-40], including hybrids of fish [26,27]. In general, the data imply a general trend of possessing two 5S rDNA classes in the fish genome [41], although there is also an exception of having one or three 5S rDNA tandem arrays [26,27]. In the hybrid offspring, 2nGB and 3nGB hybrids not only inherited two types of 5S rDNA classes (type I and II) from their parents (GC and BSB), but also generated a novel chimera of type I and type II (Figure 6B, C), which indicating that there was an occurrence of somatic recombination. In addition, these two types of 5S rDNA classes in 2nGB and 3nGB hybrids maintained an almost identical sequence to that of their parents. All the 5S rDNA sequences analyzed here were functional genes, based on the observation of ICRs (A box, IE and C box), TATA-like control element and thymidine residues (Figure 5).

It has been reported that a drastic recombination and elimination of the parental-specific DNA repeats has some relationship to the fertility of newly formed hybrids or allopolyploids [26,27,42-44]. In this study, 2nGB and 3nGB hybrids not only both inherited type I and type II of 5S rDNA from their parents, respectively, but also generated a chimera of type I and type II. In addition, a 10 bp polyA insertion in the type II sequence of chimera in 2nGB hybrids and its absence in 3nGB hybrids was also detected. We speculate that, to improve fertility, a polyA insertion in the type II sequence of chimera in 2nGB hybrids is required, though there was no direct data available to allow interpretation of the possible relationship between them.

Hybridization is thought to facilitate adaptive radiation and speciation in plants and animals [3]. It can result in alterations of gene expression, chromosomal structure, and genome size [45]. In this study, one novel chimera of type I and type II in the genome of 2nGB and 3nGB hybrids (Figure 6B, C) were observed, and nucleotide variation, including insertion-deletion and constitutions, in the NTS regions of the hybrid offspring (Figure 5B) was also detected. Taken together, these observations support the effect of hybridization and polyploidization on the organization and genetic variation of 5S rDNA multigene families, leading to rapid genomic changes.

## Conclusions

This is the first report of diploid and triploid hybrids being obtained through distant hybridization between GC and BSB which possessing the same chromosome numbers. The formation of 2nGB and 3nGB hybrids has potential benefit in aquaculture, and they can be used as a model to test theories about the origin and consequences of polyploidization. In addition, analysis of organization and variation of the 5S rDNA multigene families further revealed the influence of hybridization and polyploidization on the genome of the hybrid offspring.

## Methods

### Animals and crossing

Specimens of GC and BSB were obtained from the Engineering Research Center of Polyploid Fish Breeding and Reproduction of State Education Ministry at Hunan Normal University. During the reproductive seasons (from May to June each year) in 2008, 2009, 2010, 2011 and 2012, 10 mature female GC and 10 mature male BSB were chosen as the maternal and paternal parents, respectively. The mature eggs of GC and the mature sperm of BSB were mixed and the embryos developed in culture dishes at water temperature ranging from 19 to 20°C. Subsequently, 2000 embryos were taken at random to determine the fertilization rate (No. of embryos at the gastrula stage/No. of eggs × 100%), the hatching rate (No. of hatched fry/No. of eggs × 100%) and the adulthood rate (No. of adults/No. of eggs × 100%). At the conclusion of the experiment, the hatched fry were transferred to a special pond for further culture.

Hereinafter, the diploid hybrids of GC♀ × BSB♂ are abbreviated as 2nGB hybrids and the triploid hybrids of GC♀ × BSB♂ as 3nGB hybrids, respectively.

### Chromosome spreads and DNA content

To determine ploidy, chromosome preparations were performed using the peripheral blood cell cultures of 10 GC, 10 BSB, 10 2nGB and 10 3nGB hybrids at 1-year of age. The chromosomes were prepared in accordance with Luo et al. (2011) [46]. The shape and number of chromosomes were analyzed under a microscope. We analyzed 200 metaphase spreads (20 metaphase spreads in each sample) for each type of fish. Preparations were examined under an oil lens at a magnification of × 330, and good-quality metaphase spreads were photographed for analysis of karyotypes. Chromosomes were classified on the basis of their long-arm to short-arm ratios according to the reported standards [47].

The DNA content of erythrocytes of GC, BSB, and their hybrid offspring was measured using a flow cytometer (cell counter analyzer, Partec). 1-2 ml of blood from the caudal vein of each individual using a syringe containing ~200-300 units of sodium heparin. The blood samples were processed following the description in Liu et al. (2007) [10]. All the samples were measured under the same conditions. To calculate the probabilities of the ratios of the DNA content of the hybrid offspring to the sum of that of GC and BSB, we used the χ2 test with Yate’s correction to test deviation from expected ratio values.

### Morphological traits

The traits of 20 GC, 20 BSB, 20 2nGB and 20 3nGB hybrids were counted and measured. The countable traits included the number of lateral scales, upper lateral scales, lower lateral scales, dorsal fins, abdominal fins and anal fins. The measurable traits included the average values of whole length (WL), body length (BL), body width (BW), head length (HL), head width (HW), tail length (TL) and tail width (TW). In addition, the average ratios of body length to whole length (BL/WL), body width to body length (BW/BL), head length to body length (HL/BL), head width to head length (HW/HL), tail width to tail length (TW/TL) and head width to body width (HW/BW) were also calculated. For both measurable and countable data, the software of SPSS was used to analyze the covariance of the data of morphological traits between two kinds of hybrid offspring and their parents.

### Measurement of growth rates and feeding ecology

In January 2011, 100 GC, 100 2nGB and 100 3nGB hybrids, which were one-year-old age, were randomly selected for culture in three similar ponds (150 m^2^) and reared under the same conditions. The mean growth rate of each group was measured over a period of one year. In addition, the feeding habits of 2nGB and 3nGB hybrids were also documented.

### Genomic DNA extraction, PCR and sequencing

Total genomic DNA of GC, BSB and their hybrid offspring was extracted from the peripheral blood cells using a phenol/chloroform extraction method, as described in Sambrook et al. (1989) [48]. A set of primers (5S P1, 5′-GCTATGCCCGATCTCGTCTGA-3′: 5S P2R, 5′- CAGGTTGGTATGGCCGTAAGC-3′) were designed and synthesized to amplify the 5S rRNA genes and their nontranscribed spacer regions directly from genomic DNA according to He et al. [27]. PCR reactions were carried out in a volume of 25 μL with approximately 20 ng of genomic DNA, 1.5 mM of MgCl_2_, 250 μM of each dNTP, 0.4 μM of each primer, and 1.25 U of Taq polymerase (TaKaRa, Dalian, China). The temperature profile was: initial denaturation step at 94°C for 5 min, followed by 25 cycles (94°C for 30 s, 60°C for 30 s, and 72°C for 1 min) with a final extension step at 72°C for 10 min. Amplification products were separated on a 1% agarose gel using TBE buffer. The DNA fragments were purified using a gel extraction kit (Sangon, Shanghai, China) and ligated into the pMD18-T vector. The plasmids were transformed into E. coli DH5a and purified. The inserted DNA fragments in the pMD18-T vector were sequenced using an automated DNA sequencer (ABI PRISM 3730, Applied Biosystems, Carlsbad, CA). To analyze sequence homology and variation among the fragments amplified from GC, BSB, 2nGB and 3nGB hybrids, the sequences were aligned with BioEdit [49] and Clustal W [50].

## Abbreviations

GC: *Ctenopharyngodon idellus*; BSB: *Megalobrama amblycephala*; 2nGB: The diploid hybrids of *Ctenopharyngodon idellus* (♀) × *Megalobrama amblycephala* (♂); 3nGB: The triploid hybrids of *Ctenopharyngodon idellus* (♀) × *Megalobrama amblycephala* (♂); RCC: *Carassius auratus* red var; CC: *Cyprinus carpio*; 4nAT: The tetraploid hybrids of *Carassius auratus* red var. (♀) × *Cyprinus carpio* (♂).

## Competing interests

The authors declare that they have no competing interests.

## Authors’ contributions

WGH LHX TLL SJL and YL conceived and designed the experiments. WGH LHX and TLL performed the experiments. WGH LHX TLL SJL JX and JH analyzed the data. WGH LHX TLL JX JW and QBQ contributed reagents materials analysis tools. WGH and SJL wrote the manuscript. All authors read and approved the final version of the paper.
